# A study on medical students' willingness to participate in academic assessment and its influencing factors under the “New Medical Science” initiative

**DOI:** 10.3389/fpsyg.2026.1898915

**Published:** 2026-08-28

**Authors:** Gu Yu

**Affiliations:** Kangda College, Nanjing Medical University, Lianyungang, Jiangsu, China

**Keywords:** assessment literacy, assessment willingness, educational assessment, New Medical Science, student participation

## Abstract

Under the “New Medical Science” initiative, the educational assessment system in medical colleges and universities is facing structural issues, such as an overemphasis on quantifiable indicators that neglects students' learning processes and outcomes, and a lack of in-depth evaluation of innovative capabilities and interdisciplinary comprehensive application skills. Enhancing students' initiative and literacy in participating in assessment, and promoting the diversification and scientific design of assessment mechanisms, have become core demands for adapting to educational assessment reform in the new era. Integrating the Theory of Planned Behavior and Self-efficacy Theory, this paper explores the relationships and interaction mechanisms among subjective norms, self-efficacy, assessment literacy, and willingness to participate in assessment. Using a stratified random sample of 248 second- to fourth-year medical students from Kangda College, Nanjing Medical University, the study employs correlation analysis, regression analysis, and mediation testing. The results show that subjective norms, self-efficacy, and assessment literacy are significantly and positively associated with students' willingness to participate in assessment. Furthermore, assessment literacy plays a partial mediating role in the impact paths of subjective norms and self-efficacy on assessment willingness. Notably, the mediating effect is slightly stronger in the self-efficacy pathway than in the subjective norms pathway, suggesting a resource-gain spiral mechanism. Targeted recommendations are proposed accordingly: focusing on assessment literacy to strengthen the cultivation of students' assessment capabilities; expanding students' opportunities for assessment participation based on accumulated experience; clarifying responsibilities to create a conducive ecosystem for academic assessment; and optimizing the construction of the academic assessment system by leveraging the effectiveness of assessment practices.

## 1 Introduction

Against the backdrop of the ongoing deep transformation of the global higher education system, educational assessment, as a core mechanism for ensuring teaching quality and optimizing the structure of talent cultivation, has become a key area of policy reform in various countries (Liu, 2024; Wen and Cui, 2020). In the context of the knowledge economy and innovation-driven development, traditional educational assessment systems have revealed significant limitations, struggling to meet the multi-dimensional demands for versatile and innovative talents. In 2020, the Central Committee of the Communist Party of China and the State Council issued the Overall Plan for Deepening Educational Evaluation Reform in the New Era, proposing the improvement of evaluation systems in higher education institutions, the construction of a long-term mechanism for fostering virtue through education, and a shift away from the overreliance on singular quantitative outcomes (Liu, 2024). As a crucial pillar for the national reserve of medical and healthcare professionals, the educational assessment system of medical colleges and universities largely determines the professional competence and humanistic qualities of future medical practitioners. The scientific rigor and fairness of this assessment mechanism directly impact the overall development of the healthcare industry and public health outcomes. Therefore, establishing a systematic, comprehensive, and fair academic assessment system to promote the balanced development of medical students' knowledge, abilities, and literacy has become an urgent task in the current educational and teaching reforms of medical institutions (Wang et al., 2020).

Currently, the academic assessment systems in local higher medical colleges and universities face numerous structural problems in practice. On one hand, the prevailing assessment model emphasizes quantifiable indicators such as “employment rates,” “further study rates,” and “hardware facilities,” while paying insufficient attention to students' learning processes and outcomes, which are direct reflections of educational quality (Wang et al., 2023). On the other hand, assessment relies excessively on external, explicit results, neglecting the deep-level experiences, reflective abilities, and comprehensive literacy developed by students during the learning process. This misalignment severely conflicts with the core tenets of the “New Medical Science” (NMS) initiative, which advocates interdisciplinary integration, innovative practice, and humanistic care. In the context of NMS, medical institutions are entrusted with the mission of cultivating multi-dimensional interdisciplinary integration capabilities, innovative thinking, and a humanistic ethos among medical professionals. However, the current assessment system lacks in-depth evaluation of students' innovative capabilities and comprehensive interdisciplinary application skills, failing to effectively support this cultivation goal (Wei and Lin, 2023; Ji and Sun, 2023). Confronted with these pressing bottleneck issues, enhancing students' initiative and literacy in participating in assessment, and promoting the diversification and scientific design of assessment mechanisms, have become core imperatives for adapting to the educational assessment reforms of the new era.

This study contributes to the literature in three ways. First, it integrates the Theory of Planned Behavior and Self-efficacy Theory to construct a mediated model explaining medical students' willingness to participate in academic assessment, extending the application of these frameworks to the specific context of medical education reform under the NMS paradigm. Second, it employs quantitative analyses, including correlation, regression, and mediation testing, to examine the interrelationships among subjective norms, self-efficacy, assessment literacy, and assessment willingness. Third, it identifies the partial mediating role of assessment literacy and reveals a slightly stronger mediating effect in the self-efficacy pathway, suggesting a potential resource-gain spiral mechanism. These findings offer transferable value for medical educators and institutional administrators seeking to enhance student engagement in assessment systems.

## 2 Literature review

### 2.1 Educational assessment reform and the New Medical Science initiative

The global higher education landscape has witnessed a paradigm shift from input-oriented to outcome-based assessment models (Guo and Liu, 2014). This transition reflects broader recognition that assessment should not merely measure learning outcomes but actively shape learning processes and student development. In China, the Overall Plan for Deepening Educational Evaluation Reform in the New Era (Ji and Sun, 2023) marked a turning point, emphasizing the need to move beyond simplistic quantitative metrics toward more comprehensive, process-oriented evaluation systems (Liu, 2024). This policy shift aligns with international trends while addressing unique challenges within China's rapidly expanding higher education system.

The New Medical Science initiative represents a distinctive response to these challenges within medical education. Launched to address the evolving demands of healthcare in the 21st century, NMS advocates interdisciplinary integration, innovative practice, and humanistic care (Wei and Lin, 2023; Ji and Sun, 2023). Unlike traditional medical education models that emphasize knowledge transmission and clinical skills, NMS requires medical professionals to possess multi-dimensional competencies including critical thinking, interdisciplinary collaboration, and patient-centered communication. This expanded competency framework necessitates corresponding transformations in assessment systems. Traditional assessment approaches—heavily reliant on standardized examinations and summative evaluations—are ill-equipped to capture these complex competencies. Consequently, developing assessment systems that can validly and reliably measure NMS-aligned competencies while simultaneously promoting student engagement has become a pressing priority.

Despite growing recognition of these challenges, empirical research on medical students'assessment participation under the NMS framework remains limited. Existing studies have primarily focused on institutional-level assessment design or faculty perspectives, leaving students' voices and behavioral intentions under examined (Yang and Hu, 2016; Liang et al., 2012). This gap is particularly problematic given that student engagement is widely recognized as a critical determinant of assessment effectiveness. Without understanding what drives medical students to actively participate in academic assessment, efforts to reform assessment systems risk being misaligned with student needs and motivations.

### 2.2 Theoretical foundations: theory of planned behavior and self-efficacy theory

The Theory of Planned Behavior (TPB) provides a robust framework for understanding and predicting human behavior across diverse domains (Ajzen, 1991). According to TPB, behavioral intention—the immediate antecedent of actual behavior—is determined by three conceptually independent constructs: attitude toward the behavior, subjective norms, and perceived behavioral control. Subjective norms refer to perceived social pressure to perform or not perform the behavior, reflecting the influence of significant others such as teachers, parents, and peers (Schifter and Ajzen, 1985). In educational contexts, subjective norms have been shown to predict various student behaviors including academic engagement, help-seeking, and participation in extracurricular activities (He et al., 2023; Wang and Yao, 2022; Wang et al., 2020). For instance, research by Liu (2024) demonstrated that perceived peer and teacher expectations significantly predict students' academic engagement and their willingness to participate in formative assessment activities within medical education settings. Similarly, studies by Wang and Yao (2022) show that organizational support promotes students' participation in school internal governance behaviors. Research by Wang et al., 2020 also points out that students' willingness to engage in scientific research is closely related to peer interaction.

Self-Efficacy Theory (Bandura, 1977; Guo and Jiang, 2008) complements TPB by emphasizing the role of domain-specific confidence in shaping behavioral choices and persistence. Self-efficacy refers to an individual's belief in their capability to successfully execute courses of action required to attain designated outcomes. In academic settings, self-efficacy influences students' task choices, effort expenditure, and persistence in the face of difficulty (Du et al., 2010). Research has found that students with higher self-efficacy often demonstrate superior academic performance (Xie and Peng, 2019). Studies by Wang and Zhou (2008) discovered that self-efficacy plays a key role in learning motivation and engagement enthusiasm (Lü et al., 2024; Sun et al., 2024). Wang et al.'s research also indicates that there is a significant positive correlation between college students' self-efficacy and their knowledge reserves in specific domains (Wang et al., 2023).

While TPB and Self-Efficacy Theory have been extensively applied in educational psychology, their integration in the context of medical students' assessment participation remains underexplored. The two theories offer complementary insights: TPB highlights the role of social-environmental factors (subjective norms) in shaping intentions, while Self-Efficacy Theory emphasizes the contribution of individual psychological resources. Integrating these perspectives may provide a more comprehensive understanding of the factors driving medical students' willingness to participate in academic assessment.

While subjective norms, self-efficacy, and assessment literacy are interrelated constructs within the educational context, they are conceptually distinct and warrant separate investigation. Subjective norms refer to the perceived external social pressures (Ajzen, 1991), whereas self-efficacy denotes internal beliefs about one's own capability (Bandura, 1977). Assessment literacy, in contrast, is a task-specific competency that encompasses the knowledge, skills, and dispositions required to effectively engage with assessment processes (Guo, 2022). Although these constructs may co-vary, they operate through different mechanisms. Subjective norms exert influence via social compliance and pressure, self-efficacy through motivation and persistence, and assessment literacy through cognitive understanding and skill mastery. Treating them as separate constructs allows us to parse out their unique and combined effects on students' willingness to participate in academic assessment, which is a theoretical contribution of this study.

### 2.3 Assessment literacy and student participation

Assessment literacy has emerged as a critical construct in contemporary educational discourse (Guo, 2022; Fan, 2023). Originally conceptualized from the perspective of teachers' assessment competencies, assessment literacy has increasingly been recognized as equally important for students. Student assessment literacy refers to the comprehensive capabilities that students should possess when participating in academic assessment, including their assessment conceptions, knowledge, skills, and dispositions (Guo, 2022). This includes understanding the purposes of assessment, knowing how to interpret and use feedback, self-assessment capabilities, and the ability to engage productively in peer assessment processes (Smith et al., 2013).

Research has demonstrated a significant positive relationship between assessment literacy and students' willingness to participate. A study by Smith et al. (2013) suggests that students' understanding of assessment purposes and mastery of feedback skills can significantly enhance their engagement enthusiasm. Further research by Yang (2019) demonstrates that improving students' assessment and reflection abilities can increase their interest in political participation. These findings suggest that when students possess high levels of assessment literacy, they place greater value on assessment activities and actively engage in them.

Despite growing attention to assessment literacy, several limitations remain in the literature. First, most existing studies have focused on general higher education contexts, with limited attention to medical education specifically. Second, assessment literacy has typically been examined as either an independent variable directly predicting outcomes or as a dependent variable influenced by instructional practices. Its potential mediating role—linking external social factors and individual psychological resources to behavioral intentions—has received insufficient empirical attention. Third, the mechanisms through which assessment literacy might differentially interact with subjective norms vs self-efficacy remain unexplored.

### 2.4 Research gaps and the present study

The preceding review reveals several important gaps that this study aims to address. First, while educational assessment reform and the NMS initiative have generated considerable policy discourse, empirical research on medical students' assessment participation remains limited. Second, although TPB and Self-Efficacy Theory have been extensively validated, their integration in the specific context of medical students' assessment willingness is lacking. Third, despite growing recognition of assessment literacy's importance, its mediating role in linking subjective norms and self-efficacy to assessment willingness has not been systematically examined.

**Figure 1:**
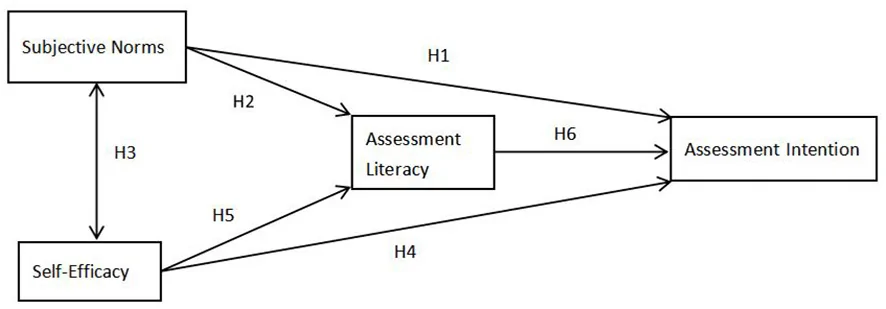
Conceptual model of factors influencing medical students' willingness to participate in academic assessment.

To address these gaps, this study constructs a mediated model examining the relationships among subjective norms, self-efficacy, assessment literacy, and medical students' willingness to participate in academic assessment. Drawing on TPB and Self-Efficacy Theory, we propose six research hypotheses (see Figure 1).

**H1:** Subjective norms have a significant positive impact on medical students' willingness to participate in academic assessment.
**H2:** Subjective norms have a significant positive impact on medical students' assessment literacy
**H3:** There is a significant correlation between subjective norms and medical students' self-efficacy.
**H4:** Self-efficacy has a significant positive impact on medical students' willingness to participate in academic assessment.
**H5:** Self-efficacy has a significant positive impact on medical students' assessment literacy.
**H6:** Assessment literacy has a significant positive impact on medical students' willingness to participate in academic assessment.

## 3 Study design and methodology

### 3.1 Research participants

A survey on willingness to participate in academic assessment was conducted among second- to fourth-year students at Kangda College, Nanjing Medical University, using stratified random sampling. To ensure data quality, the questionnaire was administered anonymously. A total of 300 questionnaires were distributed. To ensure validity, 52 questionnaires were excluded from the final analysis based on the following *a priori* criteria: (1) completion time of less than 120 s, which was considered insufficient for a thoughtful response; (2) more than 20% of the items unanswered; (3) patterned responses (e.g., selecting the same option for all items); and (4) contradictory responses to reverse-coded items. This screening process yielded 248 valid questionnaires, resulting in an effective response rate of 82.67%, leaving 248 valid questionnaires, resulting in an effective response rate of 82.67%. During the data processing stage, the collected data were first cleaned to remove obviously erroneous or inconsistent responses. Subsequently, statistical software SPSS was used for further processing, including variable coding, handling of missing values, and detection of outliers.

The first part of the questionnaire covered the characteristics of the respondents, including basic information such as gender, grade, major, and GPA. Table 1 presents the frequency analysis results for the 248 participants. In terms of gender, males accounted for 26.21%, and females accounted for 73.79%. Regarding grade distribution, the class of 2023 constituted 47.58%, the class of 2022 accounted for 33.87%, and the class of 2021 made up 18.55%. During the analysis, particular attention was paid to excluding data from first-year and fifth-year students. The reason is that first-year students generally lack sufficient course experience and professional background to effectively participate in academic assessment, while fifth-year students are more focused on internships, employment, or postgraduate entrance examinations, resulting in lower responsiveness to participating in academic assessment. Consequently, the final dataset used for analysis consisted of valid responses from second- to fourth-year students.

**Table 1:** Frequency analysis results.

Name	Option	Frequency *(n)*	Percentage (%)	Cumulative percentage (%)
Gender	Male	65	26.21	26.21
	Female	183	73.79	100.00
Grade	Class of 2023	118	47.58	47.58
	Class of 2022	84	33.87	81.45
	Class of 2021	46	18.55	100.00
Academic department	Department of Clinical Medicine	33	13.31	13.31
	Department of Nursing	28	11.29	24.60
	Department of Pharmacy	70	28.23	52.82
	Department of Public Health and Preventive Medicine	36	14.52	67.34
	Faculty of Humanities and Management	22	8.87	76.21
	Department of Medical Information Engineering	14	5.65	81.85
	Faculty of Foreign Studies	6	2.42	84.27
	Department of Rehabilitation Medicine	7	2.82	87.1
	Faculty of Medical Technology	32	12.90	100.00
Cumulative GPA	≥3.7	26	10.48	10.48
	3.4–3.69	48	19.35	29.84
	3.0–3.39	77	31.05	60.89
	2.7–2.99	54	21.77	82.66
	<2.7	43	17.34	100.00
Total		248	100.00	100.00

The distribution by academic department shows that the Department of Pharmacy had the highest number of participants at 28.23%, followed by the Department of Public Health and Preventive Medicine (14.52%) and the Department of Clinical Medicine (13.31%). Other departments, such as the Department of Nursing and the Department of Medical Technology, accounted for relatively smaller proportions. Regarding cumulative grade point average (GPA), 31.05% of students had a GPA between 3.0 and 3.39, 19.35% between 3.4 and 3.69, and 10.48% had a GPA above 3.7, indicating a certain degree of academic performance variation.

### 3.2 Research instrument

The measurement scales for the variables employed a five-point Likert scale. All measurement items were standardized, with responses scored as follows: 1 for “Strongly Disagree,” 2 for “Disagree,” 3 for “Neutral,” 4 for “Agree,” and 5 for “Strongly Agree.” Based on the research model established in this paper, which includes the four variables of assessment literacy, self-efficacy, subjective norms, and assessment intention, and grounded in the Theory of planned behavior and Self-Efficacy Theory, a certain number of measurement items were developed for each variable drawing upon relevant literature.

#### 3.2.1 Subjective norms scale

The measurement items for this dimension were developed by integrating the Theory of Planned Behavior with insights from the studies of Wang et al. (2023), Cheng (2023), and Wu and Liu (2023). The reference groups for subjective norms primarily encompass: social atmosphere (the overall societal attitude toward the decision-making behavior), organizational level (e.g., third-party independent evaluation agencies), and individual level (e.g., peers, teachers, parents). In the context of academic assessment, the items for subjective norms are designed to reveal the expectations and pressures students perceive from their surrounding environment, and how these external factors influence their decision to engage in academic assessment. A total of eight items were designed, including statements such as: “My classmates' active participation in assessment motivates me,” “I follow my teachers' suggestions regarding academic assessment,” and “The academic ethos and culture of the medical school encourage us to actively engage with academic assessment.” Higher scores indicate a stronger perception of subjective norms by the student. In this study, the Cronbach's α coefficient for this scale was 0.86, demonstrating good reliability.

#### 3.2.2 Self-efficacy scale

The measurement items for this dimension were developed by integrating Self-Efficacy Theory, the General Self-fficacy scale, the Academic Self-efficacy scale, and insights from studies by Yang and Hu (2016), Tao and Su (2022), and Wang et al. (2023). Within the context of academic assessment, self-efficacy reflects a student's confidence in their ability to successfully participate in assessment activities. The designed items aim to measure the students' psychological preparedness when facing academic challenges, as well as their anticipated capacity to utilize personal resources in problem-solving. A total of six items were designed, including statements such as: “When participating in academic assessment, I rarely withdraw due to fear of failure, ‘and' I believe I can effectively utilize feedback to improve my learning strategies” Higher scores indicate a higher level of self-efficacy in students. In this study, the Cronbach's α coefficient for this scale was 0.916, demonstrating good reliability.

#### 3.2.3 Assessment literacy scale

The design of the items for this dimension is grounded in the research of Guo (2022), Fan (2023), Yao and Yang (2023), and Ji and Sun (2023), drawing from areas such as assessment theory, assessment knowledge and skills, attitudes and values, and feedback literacy. Based on educational assessment theory—particularly contemporary assessment concepts like self-assessment, peer assessment, and formative assessment—it emphasizes that assessment serves not merely as a tool for testing knowledge, but as a crucial instrument for fostering students' self-awareness, self-management, and the development of learning strategies. The items are designed to explore students' understanding of assessment principles, mastery of assessment skills, and manifestation of assessment-related attitudes and values. The scale comprises 14 items across four sub-dimensions: (1) assessment knowledge and understanding, (2) assessment skills and application, (3) attitudes and values toward assessment, and (4) feedback literacy, which includes the ability to seek, interpret, and utilize feedback for learning improvement., including statements such as: “I understand that academic assessment aims to comprehensively understand student learning and provide developmental feedback,” “I can effectively use feedback to improve my learning strategies and performance,” “I hold an open and positive attitude toward academic assessment, viewing it as an integral part of learning,” and “I can evaluate my own learning outcomes fairly and objectively.” Higher scores indicate a higher level of assessment literacy. In this study, the Cronbach's α coefficient for this scale was 0.903, demonstrating good reliability.

#### 3.2.4 Assessment intention scale

The items for this dimension were developed by integrating the Theory of Planned Behavior with the research of Li and Sun (2022) and Wang (2022). The design of the items aims to measure students' proactivity and willingness to engage in academic assessment. A total of five items were included, such as “I am willing to participate in various forms of academic assessment to evaluate my learning outcomes.” Higher scores indicate a stronger intention to engage in assessment. In this study, the Cronbach's α coefficient for this scale was 0.949, demonstrating high reliability. To assess the construct validity of the scale, a confirmatory factor analysis (CFA) was performed using AMOS 21.0. The measurement model demonstrated a good fit to the data: χ^2^*/*df = 2.31, CFI = 0.94, TLI = 0.92, RMSEA = 0.073, and SRMR = 0.061. All factor loadings for the 14 items on their respective sub-dimensions were statistically significant (*p* < 0.001), providing evidence of acceptable convergent validity. Furthermore, the composite reliability (CR) for the four sub-dimensions ranged from 0.82 to 0.91, and the average variance extracted (AVE) ranged from 0.55 to 0.65, all exceeding the recommended thresholds, thus confirming the scale's construct validity.

### 3.3 Data processing

A database was established using EpiData 3.1 (The EpiData Association, Odense, Denmark) software. SPSS 20.0 (IBM Corp., Armonk, NY, USA) and AMOS 21.0 (IBM Corp., Armonk, NY, USA) were employed for statistical analysis. The reliability of the questionnaire was tested using Cronbach's α coefficient, while its validity was examined through exploratory and confirmatory factor analyses. The five-level weighting method for satisfaction calculation and general descriptive statistical analysis were adopted to analyze students' intention to participate in academic assessment. Correlation analysis and regression analysis were utilized to explore the interrelationships and mechanisms among subjective norms, self-efficacy, assessment literacy, and assessment intention, based on the Theory of Planned Behavior and Self-Efficacy Theory. The PROCESS macro (Version 4.0, Model 4; Andrew F. Hayes, The Ohio State University, Columbus, OH, USA) developed by Hayes (2018) was employed to test for mediating effects, using a 95% bias-corrected bootstrap confidence interval with 5,000 resamples.

## 4 Empirical analysis

### 4.1 Test for common method bias

Procedurally, the questionnaire employed methods such as anonymous responses and reverse scoring for some items, which may potentially introduce common method bias. Therefore, it is necessary to test for common method bias in the collected data using Harman's single-factor test. The results showed that four common factors with eigenvalues greater than 1 were extracted, and the variance explained by the first factor was 22.79%, which is below the 40% threshold. This indicates that no significant common method bias exists in this study.

### 4.2 Correlation analysis

As shown in Table 2, subjective norms are significantly and positively correlated with self-efficacy, assessment literacy, and assessment intention, with correlation coefficients of 0.510, 0.663, and 0.664, respectively (all *p*-values <0.05). Self-efficacy is also significantly and positively correlated with both assessment literacy and assessment intention, with correlation coefficients of 0.646 and 0.664, respectively (both *p*-values <0.05). Furthermore, assessment literacy shows a significant positive correlation with assessment intention, with a correlation coefficient of 0.696 (*p*-value <0.05). These preliminary findings provide empirical support for the hypothesized relationships.

**Table 2:** Correlation matrix.

Variables	*M*	*SD*	Subjective norms	Self-efficacy	Assessment literacy	Assessment intention
Subjective norms	3.749	0.737	1			
Self-efficacy	3.623	0.769	0.510^**^	1		
Assessment literacy	3.879	0.716	0.663^**^	0.646^**^	1	
Assessment intention	3.86	0.778	0.664^**^	0.630^**^	0.696^**^	1

^*^p < 0.05.

^**^p < 0.01.

To assess the potential for multicollinearity among the predictor variables, we examined the variance inflation factor (VIF) values. The VIF values for subjective norms, self-efficacy, and assessment literacy were all well below the commonly recommended threshold of 5.0, indicating that multicollinearity did not pose a significant threat to the stability of our regression estimates.

### 4.3 Regression analysis

This study employed the PROCESS macro developed by Hayes to test for mediating effects. After controlling for gender, grade, major, and grade point average (GPA), the mediating effect of assessment literacy was examined. As shown in the regression analysis in Table 3, in Model 1, none of the control variables had a significant effect on students' assessment intention. The independent variables, subjective norms (*B* = 0.496, *p* < 0.01) and self-efficacy (*B* = 0.411, *p* < 0.01), significantly predicted students' assessment intention. Model 2 presents the regression analysis results with the mediator as the dependent variable, showing that subjective norms (*B* = 0.433, *p* < 0.01) and self-efficacy (*B* = 0.376, *p* < 0.01) significantly predicted students' assessment literacy. The final Model 3 revealed that students' subjective norms (*B* = 0.337, *p* < 0.01), self-efficacy (*B* = 0.273, *p* < 0.01), and assessment literacy (*B* = 0.367, *p* < 0.01) all had a significant impact on their assessment intention. Furthermore, among the control variables, students' GPA also significantly and positively predicted their assessment intention (*B* = 0.063, *p* < 0.05).

**Table 3:** Regression analysis results (*n* = 248).

Variables	Model 1: assessment intention				Model 2: assessment literacy				Model 3: assessment intention			
	*B*	SE	*p*	β	*B*	SE	*p*	β	*B*	SE	*p*	β
Constant	0.277	0.29	0.341		1.186^**^	0.262	0	-	−0.158	0.286	0.581	-
Gender	0.001	0.078	0.991	0.001	−0.015	0.071	0.83	−0.009	0.006	0.074	0.930	0.004
Grade	0.031	0.045	0.488	0.030	−0.055	0.04	0.177	−0.058	0.051	0.043	0.229	0.05
Major	0.001	0.014	0.961	0.002	0.006	0.012	0.612	0.022	0.002	0.013	0.899	−0.005
GPA	0.047	0.028	0.098	0.074	−0.044	0.026	0.084	0.076	0.063^*^	0.027	0.02	0.1
Subjective norms	0.496^**^	0.053	0.000	0.47	0.433^**^	0.048	0.000	0.446	0.337^**^	0.058	0.000	0.319
Self-efficacy	0.411^**^	0.052	0.000	0.406	0.376^**^	0.047	0.000	0.404	0.273^**^	0.055	0.000	0.27
Assessment literacy									0.367^**^	0.067	0.000	0.337
*R* ^2^	0.562				0.577				0.61			
Adjusted *R*^2^	0.551				0.567				0.599			
*F*	*F*_(6, 241)_= 51.541, *p* < 0.001				*F*_(6, 241)_ = 54.810, *p* < 0.001				*F*_(7, 240)_ = 53.641, *p* < 0.001			

^*^p < 0.05.

^**^ *p* < 0.01.

The reference groups were set as follows: female for gender, sophomore (second-year) for academic year, and Clinical Medicine for major.

Before conducting the mediation analysis, we performed a series of regression analyses to examine the direct effects among the variables. As shown in Table 3, Model 1, which included control variables (gender, grade, major, and GPA) and the independent variables (subjective norms and self-efficacy), explained a significant proportion of the variance in assessment intention [*R*^2^ = 0.562, Adjusted *R*^2^ = 0.551, *F*_(6, 241)_ = 51.541, *p* < 0.001]. Model 2, which regressed assessment literacy on the same set of predictors, demonstrated an *R*^2^ of 0.577 [Adjusted *R*^2^ = 0.567, *F*_(6, 241)_ = 54.810, *p* < 0.001]. The final Model 3, which included all predictors and the mediator (assessment literacy), exhibited the highest explanatory power, accounting for 61.0% of the variance in assessment intention [*R*^2^ = 0.610, Adjusted *R*^2^ = 0.599, *F*_(7, 240)_ = 53.641, *p* < 0.001]. These results indicate that the proposed model provides a good fit to the data and that the inclusion of assessment literacy as a mediator substantially improves the prediction of students' willingness to participate in academic assessment.

### 4.4 Mediation analysis

According to the mediation test results presented in Table 4, the table displays the results of the mediation effect analysis for the impact of “subjective norms” and “self-efficacy” on “assessment intention” through “assessment literacy.” Both pathways demonstrate a partial mediation effect, indicating that “assessment literacy” plays a partial explanatory role between the independent and dependent variables, while also showing significant direct effects.

**Table 4:** Mediation analysis results.

Item	Effect size	95% CI		SE	*z/t*	*p*
		Lower limit	Upper limit			
Subjective norms → assessment literacy → assessment intention	0.159	0.068	0.244	0.045	3.536	0
Subjective norms → assessment literacy	0.433	0.338	0.527	0.048	8.966	0
Assessment literacy → assessment intention	0.367	0.234	0.499	0.067	5.438	0
Subjective norms → assessment intention	0.337	0.223	0.451	0.058	5.784	0
Self-efficacy → assessment literacy → assessment intention	0.138	0.078	0.195	0.031	4.497	0
Self-efficacy → assessment literacy	0.376	0.284	0.467	0.047	8.064	0
Assessment literacy → assessment intention	0.367	0.234	0.499	0.067	5.438	0
Self-efficacy → assessment intention	0.273	0.165	0.381	0.055	4.97	0

In the “subjective norms → assessment intention” pathway, the total effect is 0.496, the indirect (mediated) effect is 0.159, and the direct effect is 0.337. The mediation effect accounts for 31.98% of the total effect. In the “self-efficacy → assessment intention” pathway, the total effect is 0.411, the indirect (mediated) effect is 0.138, and the direct effect is 0.273. The mediation effect accounts for 33.53% of the total effect. This indicates that the mediating role of “assessment literacy” is slightly stronger in the “self-efficacy” pathway than in the “subjective norms” pathway.

## 5 Discussion

This study aimed to investigate the interplay between subjective norms, self-efficacy, assessment literacy, and medical students' willingness to participate in academic assessment within the context of the NMS initiative. The results confirmed our hypothesized model, revealing that subjective norms and self-efficacy not only directly and positively influence assessment willingness but also indirectly affect it through the partial mediation of assessment literacy. While these findings generally align with the theoretical predictions of the Theory of Planned Behavior (Ajzen, 1991) and Self-Efficacy Theory (Bandura, 1977), a deeper discussion reveals both consistencies and unique contributions compared to prior research.

First, the significant direct effects of subjective norms and self-efficacy on assessment willingness (supporting H1 and H4) are consistent with a broad range of studies in educational psychology (He et al., 2023; Du et al., 2010). However, the effect size of self-efficacy is comparable to, yet slightly different from, findings in non-medical disciplines. For instance, studies in general higher education often report self-efficacy as the dominant predictor of academic engagement. In our medical student sample, subjective norms exerted a slightly stronger influence. This subtle but important difference may be attributed to the unique, high-stakes, and collectivistic learning environment of medical education in China. Medical students are often socialized into a rigorous, hierarchical culture where the expectations of clinical teachers, resident mentors, and even peers carry substantial weight. The perceived pressure to meet these professional standards (“subjective norms”) appears to be a particularly potent motivator for their participation in academic assessment, potentially superseding even their own self-confidence in specific contexts.

Second, the mediating role of assessment literacy is a key finding. While the mediating effect was significant for both paths, its proportion was slightly larger in the self-efficacy → assessment intention pathway than in the subjective norms → assessment intention pathway. This nuanced difference provides a more refined understanding than previous studies that simply confirmed a mediating effect (e.g., Smith et al., 2013). Our result suggests that assessment literacy plays a slightly more critical role in translating internal psychological states (self-efficacy) into action than in responding to external pressures (subjective norms). A plausible explanation is that students with high self-efficacy are more likely to proactively seek out and master assessment-related knowledge and skills (e.g., understanding criteria, using feedback), as this mastery further reinforces their confidence. In contrast, a student primarily driven by external norms might participate actively but with a more surface-level engagement, not necessitating a deep enhancement of their underlying assessment literacy. This “gain spiral” mechanism, well-documented in Conservation of Resources theory (Wang and Yao, 2022), highlights how an initial personal resource (self-efficacy) can be leveraged to acquire a task-specific resource (assessment literacy), which then collectively boosts the target behavior. This finding extends the application of TPB by specifying a key psychological mechanism—the accumulation of competence—that is not fully explicated in the original model.

Third, the role of GPA as a significant control variable in the final model is noteworthy. This indicates that academically stronger students are more willing to participate in assessments, a finding that diverges from some studies that report a non-significant or even negative relationship. This might be specific to the context of “New Medical Science,” where assessments are evolving to be more comprehensive and challenging. Higher-achieving students may perceive these new forms of assessment not as a threat, but as an opportunity to further distinguish themselves and demonstrate their multidisciplinary competencies, thereby exhibiting a stronger willingness.

The findings contribute to the existing literature in several ways. Theoretically, this study demonstrates the value of integrating TPB and Self-Efficacy Theory to understand medical students' assessment participation, revealing how external social factors (subjective norms) and internal psychological resources (self-efficacy) jointly shape behavioral intentions through the partial mediation of assessment literacy. The identification of a slightly stronger mediating effect in the self-efficacy pathway provides initial evidence for a resource-gain spiral mechanism that warrants further investigation. Methodologically, this study validates a set of reliable instruments for measuring subjective norms, self-efficacy, assessment literacy, and assessment intention in the Chinese medical education context.

## 6 Conclusion and implications

### 6.1 Conclusion

This study concludes that subjective norms, self-efficacy, and assessment literacy are significant positive predictors of medical students' willingness to participate in academic assessment within the context of the New Medical Science initiative in China. Specifically, students who perceive stronger support from teachers, peers, and the broader academic environment, and who possess greater confidence in their own capabilities, are more likely to demonstrate a proactive intention to engage in assessment activities. More importantly, assessment literacy acts as a partial mediator in the relationships between both subjective norms and self-efficacy and the outcome variable. This mediating role reveals that external social pressures and internal psychological resources not only directly shape willingness but also operate through students' understanding, skills, and attitudes toward assessment. Notably, the mediating effect is slightly stronger in the self-efficacy pathway than in the subjective norms pathway, suggesting a resource-gain spiral in which initial confidence facilitates the acquisition of assessment literacy, which in turn further enhances participation willingness. These findings extend the application of the Theory of Planned Behavior and Self-Efficacy Theory to the specific domain of medical education reform.

### 6.2 Practical implications

The following practical implications are derived from the empirical associations and predictive models identified in this study. They should be viewed as theory-guided strategies for enhancing student engagement, rather than as evidence-based prescriptions from a controlled intervention. Future research is needed to test the effectiveness of these proposed strategies through rigorous experimental or quasi-experimental designs.

The findings offer several targeted practical implications for medical educators, institutional administrators, and assessment system designers under the NMS paradigm.

#### 6.2.1 Focusing on assessment literacy to strengthen the cultivation of students' assessment capabilities

Fostering students' assessment literacy should become a central pedagogical goal. Rather than treating assessments merely as grading tools, educators should explicitly demystify assessment criteria, provide actionable and timely feedback, and integrate self-assessment and peer-assessment training into the curriculum. When students understand the purposes, processes, and standards of assessment, they are more likely to perceive it as a developmental opportunity rather than a threat, thereby increasing their engagement.

#### 6.2.2 Expanding students' opportunities for assessment participation based on accumulated experience

Targeted interventions should be designed for students with lower academic self-efficacy or those from less supportive normative environments. Mentorship programs that pair less confident students with experienced peers or faculty, peer-assisted learning groups, and mastery-oriented feedback that emphasizes effort and improvement can effectively boost self-efficacy beliefs.

#### 6.2.3 Clarifying responsibilities to create a conducive ecosystem for academic assessment

Faculty development initiatives should encourage teachers to create a more supportive, collaborative, and open classroom climate, where positive subjective norms—such as peer encouragement and teacher recognition—can be leveraged to motivate participation.

#### 6.2.4 Optimizing the construction of the academic assessment system by leveraging the effectiveness of assessment practices

Assessment system designers should move beyond traditional outcome-focused metrics. Incorporating low-stakes formative assessments, providing second-chance opportunities for improvement, and ensuring transparency and consistency in grading criteria can significantly reduce student anxiety and promote a sense of ownership, fairness, and psychological safety. These conditions are essential for cultivating the sustained willingness to participate that the NMS paradigm demands, ultimately supporting the development of well-rounded, innovative medical professionals.

### 6.3 Limitations and future research

This study has several limitations that should be acknowledged. First, the data are cross-sectional, which fundamentally limits our ability to draw causal inferences about the relationships among subjective norms, self-efficacy, assessment literacy, and assessment willingness. For example, it is equally plausible that students who are more engaged in assessment activities achieve higher self-efficacy, rather than higher self-efficacy leading to greater willingness to participate. Future research should employ longitudinal or experimental designs to clarify the direction and temporal sequence of these relationships. Second, the sample was drawn from a single medical college, which may limit the generalizability of the findings to other institutional contexts. Future studies could expand the sampling frame to include multiple medical institutions across different regions of China. Third, the study relied exclusively on self-report measures, which may be subject to social desirability bias and common method variance. While Harman's single-factor test suggested that common method bias was not a significant concern, self-report data may not perfectly correspond to students' actual assessment literacy or their real-world participation behaviors. Future research could strengthen the validity of these findings by incorporating objective measures of assessment performance, such as analyzing students' actual assessment scores or tracking their voluntary participation in assessment-related activities. Multi-informant designs, such as including peer or teacher evaluations of a student's engagement, could also provide a more holistic and less biased assessment. Fourth, while our findings revealed a slightly stronger mediating effect for the self-efficacy pathway, the study did not directly test the resource-gain spiral mechanism. Future research could examine this mechanism more directly using experimental or longitudinal designs. Fifth, the exclusion of first-year and fifth-year students, while methodologically justified to ensure the relevance and responsiveness of the participants, may limit the generalizability of the findings to these specific cohorts at the beginning and end of their medical training. Sixth, the participant distribution across academic departments was uneven, with the Department of Pharmacy contributing a substantially larger proportion of respondents. This imbalance may influence the observed relationships and reduce the applicability of the results to the broader medical student population across all disciplines.

Despite these limitations, this study provides a foundation for understanding medical students‘ willingness to participate in academic assessment under the NMS initiative. By illuminating the roles of subjective norms, self-efficacy, and assessment literacy, the findings offer both theoretical insights and practical guidance for enhancing student engagement in medical education assessment systems.

## Data Availability

The original contributions presented in the study are included in the article/supplementary material, further inquiries can be directed to the corresponding author/s.
